# Pattern of microRNA expression associated with different stages of alcoholic liver disease in rat models

**DOI:** 10.3892/mmr.2014.2368

**Published:** 2014-07-07

**Authors:** YI-PENG CHEN, XI JIN, MEI KONG, YOU-MING LI

**Affiliations:** 1 Department of Gastroenterology, The First Affiliated Hospital, Zhejiang University, Hangzhou, Zhejiang 310003, P.R. China; 2 Department of Pathology, The First Affiliated Hospital, Zhejiang University, Hangzhou, Zhejiang 310003, P.R. China

**Keywords:** microRNA, alcoholic liver disease, microarray, stem-loop quantitative polymerase chain reaction

## Abstract

Emerging evidence has suggested that aberrant expression of micro (mi)RNAs contributes to the development of alcoholic liver injury (ALD). However, miRNA profiles distinguishing different stages of ALD have not yet been reported. The present study was designed to investigate the unique miRNA expression patterns at different stages of ALD in a rat model and analyze the gene functions and pathways of dysregulated miRNA-targeted genes. Using microarray and stem-loop quantitative polymerase chain reaction analyses, 16 miRNAs were identified as upregulated and 13 were identified as downregulated in an alcoholic steatohepatitis (ASH) group compared with the control group, while five miRNAs were identified to be upregulated and eight were identified to be downregulated in the alcoholic fatty liver (AFL) group as compared with the control group. Following further confirmation by Significance Analysis of Microarray and prediction by Prediction Analysis of Microarray, 8 and 12 types of miRNA were screened as molecular signatures in distinguishing AFL and ASH, respectively, from normal rat liver. In addition, several miRNA-target pairs were predicted by computer-aided algorithms (Kyoto Encyclopedia of Genes and Genomes pathway enrichment analyses using the Database for Annotation, Visualization and Integrated Discovery platform) and these genes may be involved in cancer signaling pathways, the Wnt signaling pathway and other signaling pathways. These results may provide novel miRNA targets for diagnosis and therapeutic intervention at different stages of ALD.

## Introduction

Alcoholic liver disease (ALD) is a predominant cause of chronic liver disease worldwide, encompassing a wide spectrum of liver damage, including simple steatosis of alcoholic fatty liver (AFL), alcoholic steatohepatitis (ASH), fibrosis, cirrhosis and superimposed hepatocellular carcinoma (1). Although steatosis is an almost completely benign disease, liver cirrhosis is associated with notable morbidity and mortality, with a shortened life expectancy. One surveillance report revealed that liver cirrhosis was the twelfth leading cause of mortality in the United States in 2007, with 48% of cirrhosis-related mortality associated with alcohol use (2). According to the Chinese guidelines on ALD for 2010, the prevalence of ALD was 3.94% in Zhejiang province in 2010 (3). Therefore, developing effective diagnostic tools and treatments for ALD is of great clinical importance.

Micro (mi)RNAs are non-coding sequences of 19–25 nucleotide length that bind to the 3′-untranslated region of target transcripts and regulate gene expression by degradation of target mRNAs or inhibition of translation (4,5). Emerging evidence suggests that aberrant expression of miRNAs contributes to the development of alcoholic liver injury. For example, Tang *et al* (6) reported that alcohol-induced overexpression of miR-212 resulted in gut leakiness by downregulation of a major component of tight junctions, Zonula occludens 1. Gut leakiness is a key factor in alcoholic liver disease. Yin *et al* (7) demonstrated that chronic exposure to ethanol markedly and specifically induced miR-217 overexpression in AML-12 hepatocytes and in mouse livers, which then inhibited hepatic sirtuin 1 expression and ultimately resulted in fat accumulation in hepatocytes. Treatment of normal human hepatocytes and cholangiocytes with ethanol induced a significant increase in miR-34a expression levels. Overexpression of miR-34a reduced ethanol-induced apoptosis by targeting caspase-2 and sirtuin 1 (8). miR-155 and miR-132 expression levels were also demonstrated to be increased in the total liver as well as in isolated hepatocytes and the Kupffer cells of alcohol-fed mice (9).

Other studies have also analyzed miRNA profiles in alcoholic liver disease by microarray. A total of 26 microRNAs were identified in ALD patients by Liu *et al* (10). Further microRNA-gene networks indicated that the key microRNAs were *Homo sapiens* (hsa)-miR-570, hsa-miR-122, hsa-miR-34b, hsa-miR-29c, hsa-miR-922 and hsa-miR-185, which negatively regulated ~79 downstream target genes to modulate hepatocyte immune response, inflammatory response and glutathione metabolism (10). Chronic ethanol feeding was observed to alter the expression levels of several miRNAs during liver regeneration, including miR-34a, miR-103, miR-107, miR-122 (11) and miR-21 (12). Dolganiuc *et al* (13) demonstrated that the Lieber-deCarli alcohol diet upregulated 1% of known miRNAS, including miR-705 and miR-1224, and downregulated 1% of known miRNAs, including miR-182, miR-183 and miR-199a-3p in mice with alcoholic steatohepatitis compared with pair-fed controls. These findings provide insight into possibilities for the diagnosis and treatment of ALD. However, the miRNA profiles distinguishing ASH from AFL (different stages of ALD) have, to the best of our knowledge, not yet been reported.

In the present study, the miRNA expression patterns at different stages of ALD were investigated by microarray analysis and stem-loop quantitative polymerase chain reaction (qPCR) analysis of liver tissues from a rat model. The successful distinction of AFL and ASH from normal liver may support the argument for using miRNA profiles as a novel diagnostic tool for ALD. In addition, the targets of miRNAs were predicted with computer-aided algorithms (TargetScan, miRanda and PicTar) and the underlying functions of these target genes were analyzed by Gene Ontology (GO) categories and Kyoto Encyclopedia of Genes and Genomes (KEGG) pathway enrichment. Unique miRNAs may be important at different stages of ALD by influencing the expression levels and altering the functions of target genes.

## Materials and methods

### Experimental animal model, serological and pathological markers

A total of 48 Sprague-Dawley rats, aged 12 weeks, weighing 160–170 g, purchased from the Medical Science Institution of Zhejiang Province (Hangzhou, China), were randomly divided into four groups: C12 (control, 12 weeks, n=12), AFL (simple steatosis, 12 weeks, n=12), C16 (control, 16 weeks, n=12) and ASH (alcoholic steatohepatitis, 16 weeks, n=12). All rats were maintained on a 12-h light/dark cycle and provided *ad libitum* food and water throughout the experiment. Rats in the AFL and ASH groups were gavaged with Chinese distillate spirit (56% alcohol administered as 17.86 ml/kg once per day; Erguotou, Red Star, Beijing, China), while those in the control groups were administered saline (14). At the end of either 12 or 16 weeks, the rats were sacrificed by femoral exsanguination under deep ether anesthesia. The levels of serum alanine aminotransferase (ALT), aspartate aminotransferase (AST), triglyceride (TG), gamma-glutamic transpeptidase (GGT), total cholesterol (TCh) and hepatic TG were routinely determined. Liver paraffin sections were stained with hematoxylin and eosin (H&E, Sigma Chemical Co., St. Louis, MO, USA) and examined under a microscope (Olympus BX41; Olympus Optical Co., Ltd., Tokyo, Japan). The severity of hepatic injury was determined by the histological activation index (HAI) as previously described (15). This protocol was approved by the Review Board at Zhejiang University and The First Affiliated Hospital (Hangzhou, China). All animal studies were performed in accordance with the guidelines of the institution for the care and use of experimental animals.

### Microarray and stem-loop qPCR

Total RNA enriched with miRNAs was extracted from liver tissues using the *mir*Vana^™^ miRNA Isolation kit (Ambion, Austin, TX, USA) and subjected to direct labeling with an Hy3^™^ fluorescent label using the miRCURY^™^ Array Labeling kit (Exiqon, Vedbæk, Denmark). Subsequent to labeling, the RNA was concentrated with the RNeasy Mini kit (Qiagen, Hilden, Germany) followed by hybridization using the miRCURY^™^ Array Microarray kit (Exiqon, Vedbaek, Denmark). The hybridized arrays were scanned with the Genepix 4000B scanner (Axon Instruments, Sunnyvale, CA, USA) and the microarray images were background-subtracted, normalized and subjected to further analysis. Mature miRNAs with comparative expression levels of >2.0 or <0.5 between AFL and C12, or ASH and C16 were considered as dysregulated miRNAs.

To further validate the expression levels of significantly dysregulated miRNAs revealed by microarray, stem-loop qPCR was performed with stem-loop antisense primer mix (Applied Biosystems, Foster City, CA, USA) and Avian Myeloblastosis Virus transcriptase (Takara Bio, Inc., Dalian, China). All PCR primers were designed based on miRNA sequences released by the Sanger Institute (16). The relative quantity of each miRNA was normalized to U6 RNA and calculated using the following equation: 2^−ΔCT^, where ΔC_T_ = C_TmiRNA_-C_TU6_.

### Bioinformatical analysis of miRNAs with significant differential expression

The raw -ΔC_T_ values from the stem-loop qPCR results were normalized, mean-centered, log_2_-transformed and jointly analyzed by Significance Analysis of Microarray (SAM, http://www-stat.stanford.edu/~tibs/SAM/) (17) and Prediction Analysis of Microarrays (PAM, http://www-stat.stanford.edu/~tibs/PAM/) (18) that were developed at Stanford University Labs and available free of charge. SAM was used to identify miRNAs that exhibited significant differences in expression levels between the disease stages. miRNAs with >2-fold changes and false discovery rate (FDR, q) <0.05 were considered as significantly regulated. PAM was then used to construct a classifier to determine the miRNA signatures at the different disease stages as determined by training, take-one-out cross validation and testing procedures. Hierarchical clustering of the miRNAs (from SAM results) and samples (from AFL and ASH) via the algorithm of average linkage and Euclidian distance (19) was performed to visualize the numerical changes through graphical presentation of the raw -ΔC_T_ values.

### Computational analysis of the functions and pathways of miRNA downstream targets

Downstream target prediction was conducted mainly according to the workflow developed by Masotti and Alisi (20). Target genes of PAM-selected miRNAs were predicted by three bioinformatical algorithms: TargetScan (http://www.targetscan.org) (21), miRanda (www.microrna.org) (22) and PicTar (pictar.bio.nyu.edu) (23), where only those genes predicted by all algorithms were selected. Any gene predicted by up- and downregulated miRNAs simultaneously was removed. In addition, genes commonly targeted by all upregulated or downregulated miRNAs at the ASH or AFL stages were predicted by the ‘microRNA’ (http://www.microrna.org/microrna/home.do) platform. The function and network of those common targeted genes were analyzed using the GO and KEGG pathway from the Database for Annotation, Visualization and Integrated Discovery (http://david.abcc.ncifcrf.gov/), where FDR and P-value were computed. The smaller the FDR, the smaller the error in calculating the P-value.

### Statistical analysis

Each experiment was performed in triplicate. All data were analyzed with the SPSS 17.0 software package (SPSS Inc., Chicago, IL, USA) and expressed as the mean ± the standard error of the mean. Student’s t-test was used for comparisons between two unpaired groups, while one-way analysis of variance was employed for comparisons among three groups. P<0.05 was considered to indicate a statistically significant difference.

## Results

### Establishment of the ALD rat model

**Figure 1:**
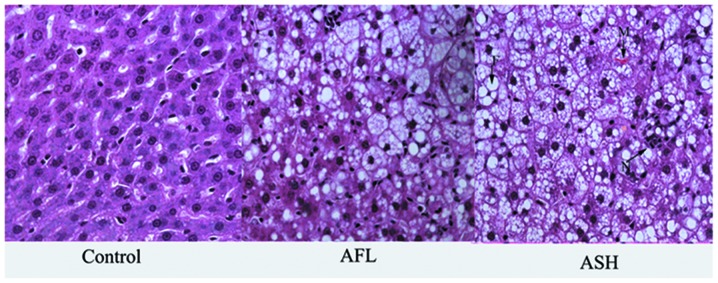
Hematoxylin and eosin staining of liver sections exhibiting the pathological features of hepatic steatosis and steatohepatitis. AFL, simple steatosis group; ASH, alcoholic steatohepatitis group.

Different stages of the ALD rat model were successfully established, as confirmed by serological and pathological changes. Compared with the control groups, the expression levels of serum TG, TCh and GGT were significantly increased in the AFL and ASH groups, although the HAI score, and the ALT and AST expression levels were only significantly enhanced at the ASH stage (P<0.05, Table I). In addition, the hepatic TG expression levels and the hepatic index were significantly increased at the AFL and ASH stages compared with the respective control groups (P<0.05); however, the differences in these measurements between the two disease stages were not identified as statistically significant. H&E staining of liver tissue revealed hepatic lipid accumulation (predominantly macrovesicular) after 12 weeks of alcohol gavage. In the ASH group, more severe steatosis and various degrees of chronic inflammation were detected in the portal area and acinar zone 3, including mallory bodies and inflammatory cell infiltration (Fig. 1).

### Identification of miRNA expression patterns at different stages of ALD

The majority of the miRNAs identified by microarray as significantly dysregulated were verified as dysregulated by stem loop qPCR, with the exception of *Rattus norvegicus* (rno)-miR-344a/rno-miR-344a-5p, rno-miR-3572, rno-miR-138-1, rno-miR-3596c, rno-let-7i and rno-miR-465, providing evidence that the microarrays effectively screened dysregulated miRNAs. Compared with the control group, 16 miRNAs were upregulated, while 13 miRNAs were downregulated in the ASH group, where miR-129 and miR-199a-3p exhibited the greatest upregulation and downregulation, respectively, of the miRNAs. Five upregulated and eight downregulated miRNAs were observed in the AFL group compared with the expression levels in the control group, where miR-200c and miR-93 exhibited the greatest upregulation and downregulation, respectively, of the miRNAs. Among the dysregulated miRNAs, miR-490 exhibited an increment in the ASH and AFL groups, while miR-140, miR-7a, miR-451, miR-93, miR-146a and miR-191 levels exhibited a decline in the ASH and AFL groups compared with the respective controls (Table II). No miRNAs with opposite expression patterns in the ASH and AFL groups were identified.

**Figure 2:**
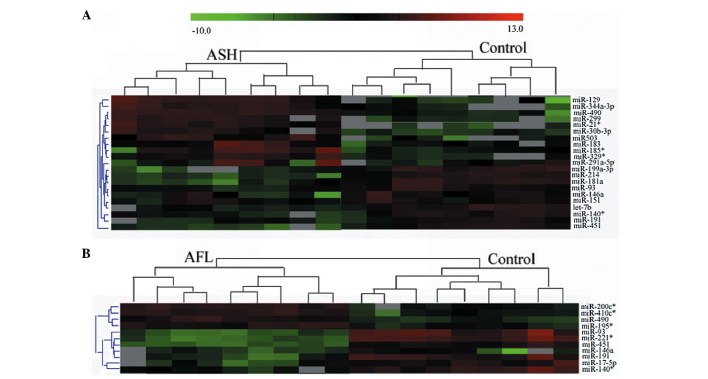
Heat map illustrating expression patterns of upregulated or downregulated microRNAs between the (A) ASH or (B) AFL group and the respective control groups. ASH, alcoholic steatohepatitis group, vs. Control, normal 16-week liver group; AFL, alcoholic fatty liver group, vs. Control, normal 12-week liver group.

In addition, SAM was utilized to identify miRNAs that were significantly associated with the two stages of ALD in comparison to the respective controls. A total of 21 (ASH versus C16) and 11 (AFL versus C12) differentially expressed miRNAs were identified (Table III). PAM was also applied for selecting molecular diagnosis markers. The unique miRNA expression patterns distinguishing the ASH group from the control group were composed of six downregulated (miR-199a-3p, miR-214, miR-93, miR-146a, miR-191 and let-7b) and six upregulated (miR-129, miR-490, miR-21, miR-503, miR-183 and miR-185) miRNAs. Similarly, the specific miRNA profile of AFL consisted of five downregulated (miR-93, miR-451, miR-221, miR-17-5p and miR-146a) and three upregulated (miR-200c, miR-490 and miR-195) miRNAs, in comparison to the control group. A heat map was constructed to visualize the results of hierarchical clustering, where a general distinction between samples of model and control groups and two major branches in rows (upregulated and downregulated miRNAs) was clearly observed (Fig. 2).

### miRNA target prediction by computer-aided algorithms

**Figure 3:**
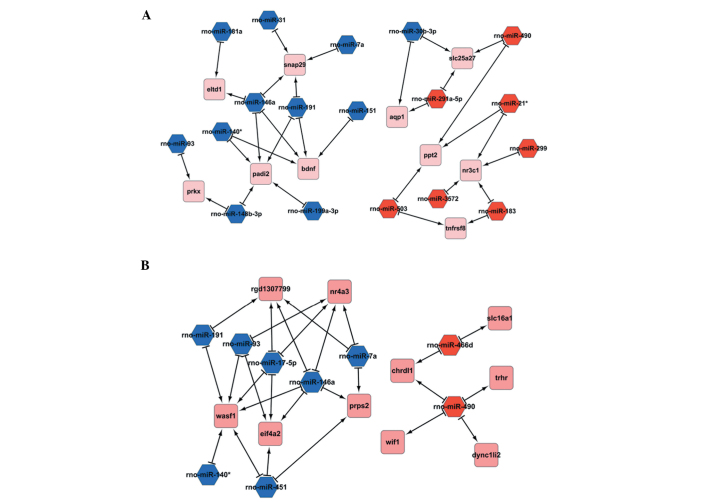
miRNA-top five target gene interaction network constructed by cytoscape software (version 2.5.1; http://www.cytoscape.org). The top five target genes were screened according to the rank of miRNA/target gene pair-specific context score. Blue indicates downregulated miRNAs, red indicates upregulated miRNAs and pink indicates the target genes. (A) Alcoholic steatohepatitis group and (B) simple steatosis group. miRNA, microRNA.

Following combined target prediction and overlapped gene deletion, a total of 2,421 and 1,327 genes were identified as targeted by downregulated and upregulated miRNAs, respectively, from the ASH group. Similarly, 2,105 and 1,651 targets of downregulated and upregulated miRNAs, respectively, were identified from the AFL group. To further narrow the gene scope and generate more information, common genes targeted by all upregulated or downregulated miRNAs from the ASH and AFL groups were predicted. In total, 8 and 61 common target genes of downregulated and upregulated miRNAs, respectively, were detected in the ASH group. A total of 20 and 152 common target genes of downregulated and upregulated miRNAs, respectively, were identified from the AFL group. According to the rank of miRNA/target gene pair-specific context score, the top five target genes were screened and presented in Table IV and the network of the interactions among miRNAs and target genes are presented in Fig. 3.

To systematically analyze this large quantity of information, GO categories and KEGG pathways were further applied. For genes targeted by all downregulated miRNAs from the ASH group, six GOTERM_BP_FAT categories were observed, including electron transport chain, phosphorylation, generation of a signal involved in cell-cell signaling, phosphate metabolic processes, phosphorus metabolic processes and cell secretion. By contrast, no common KEGG pathways were detected for those genes. For genes targeted by all upregulated miRNAs from the ASH group, 54 GOTERM_BP_FAT categories were identified, where the top five were: Small guanosine triphosphate (GTP)ase-mediated signal transduction, intracellular signaling cascade, response to organic substances, response to endogenous stimuli and cation transport (Table V). In addition, the KEGG pathways for those genes included signaling pathways in cancer, non-small cell lung cancer, pancreatic cancer, rental cell carcinoma and colorectal cancer (Table VI). Notably, no GOTERM_BP_FAT categories and KEGG pathways for genes were targeted by all downregulated miRNAs from the AFL group. For genes targeted by all upregulated miRNAs from the AFL group, 97 GOTERM_BP_FAT categories were detected, where the top five were: Cell-cell adhesion, homophilic cell adhesion, biological adhesion, cell adhesion and small GTPase-mediated signal transduction (Table V). Furthermore, eight KEGG pathways were identified for those genes, including colorectal cancer, the mitogen-activated protein kinase signaling pathway, pancreatic cancer, signaling pathways in cancer, neurotrophin signaling pathway, Wnt signaling pathway, lysosome and neuroactive ligand-receptor interaction (Table VI).

## Discussion

Previous studies have provided specific miRNA profiles for different stages of nonalcoholic fatty liver disease (15,24). The findings revealed that dysregulated miRNAs may be involved in nonalcoholic fatty liver disease progression by regulating downstream genes important in hepatocyte apoptosis, fatty acid metabolism, ion transport and membrane structure. In the present study, the same strategy (joint microarray and qPCR) was employed to screen and verify miRNA profiles at two different stages of a well-established alcoholic liver disease rat model, followed by bioinformatical analysis of downstream gene functions. The ~90% overlap between the microarray and qPCR results further supported the significance of the microarray in roughly screening candidate miRNAs. As shown in Table II, 21 (ASH versus C16) and 11 (AFL versus C12) differentially expressed miRNAs were identified through the SAM algorithm, where miR-129 and miR-199a-3p exhibited the highest degrees of upregulation and downregulation between the ASH and the respective control groups, and miR-200c and miR-93 exhibited the highest upregulation and downregulation between the AFL and the respective control group. These miRNAs have been demonstrated to be involved in a number of pivotal pathophysiological processes. For instance, miR-129 has been reported to be associated with gastric cancer (25), thyroid cancer (26) and bladder cancer (27). Such association may partially explain the increased occurrence of hepatocellular carcinoma in ASH patients. miR-199a-3p is involved in somatic cell reprogramming (28) and cell proliferation (29). Reduced miR-199a-3p levels in ASH (13) may decrease hepatocyte proliferation and invasion, resulting in a small possibility of progression from alcohol-induced steatohepatitis to hepatocellular carcinoma. Overexpression of miR-200c or miR-200b has been demonstrated to upregulate E-cadherin expression, which promotes cell adhesion and thus reduces the initiation of an invasive phenotype (30). miR-93 is able to suppress proliferation and colony formation of human colon cancer stem cells (31).

One pivotal finding of the present study was that the PAM-selected miRNA profile (8 miRNAs in the AFL group and 12 miRNAs in the ASH group) provided ~100% diagnostic accuracy of the different stages of ALD when compared with liver biopsy. This provides a rationale for further investigation of the diagnostic value of miRNA profiling in humans and may provide novel and effective molecular markers for ALD diagnosis. Furthermore, these miRNAs were hypothesized to be involved in ALD progress by interacting with the underlying target genes, of which some were enriched in GO terms or the KEGG pathway (Tables V and VI). For instance, miR-191 may regulate peptidylarginine deiminase type 2 (PAD-2) and brain-derived neurotrophic factor (BDNF). PAD is significantly associated with Metavir activity and liver fibrosis scores (32). Chronic drinking results in a reduction in BDNF levels (33) and inhibits BDNF-induced Rac1/Cdc42 activation, thus alleviating liver injury and fibrosis (34). Protein kinase, X-linked (PRKX), which was determined to be regulated by miR-93 in the present study, is a catalytic subunit of cyclic AMP-dependent protein kinases and may be important in the liver through mediation of endothelial cell proliferation, migration and vascular-like structure formation (35). MiR-183 may inhibit tumor necrosis factor receptor superfamily, member 8 (TNFRSF8, also termed CD30) expression, a marker of cells producing Th2 (T-helper-2)-type cytokines, and the Th2-type cytokine profile has been documented in ALD (36). The expression levels of nuclear receptor subfamily 3, group C, member 1 (glucocorticoid receptor, nr3c1), which was also identified to be regulated by miR-183, may be associated with alcohol abuse (37). Wiskott-Aldrich syndrome protein family member 1 (Wasf1), which was found to be regulated by miR-17-5p, miR-93, miR-191, miR-451, miR-146a and miR-140, has been shown to be a protective alcohol-responsive gene in the prefrontal cortex of human alcoholics (38). Wnt inhibitory factor 1 (Wif1), which may be regulated by miR-490, contributes to the activation of the Wnt signaling pathway and induces carcinogenesis through dysregulation of cell proliferation and differentiation (39). These findings may provide mechanisms underlying the pathogenesis and progression of ALD.

There are numerous limitations in the present study. The associations between miRNAs and the respective target genes and the underlying functions of these genes were obtained only through bioinformatical analysis. Further *in vitro* experiments are required to confirm the results. It remains controversial whether the miRNA profiles of rats may be generalized to humans. However, as certain ‘miRNA orthologs’ are identical, and the liver pathological changes are similar in ALD rats and humans, the results may be applicable in humans. As expected, several miRNAs, including miR-185 (10) and miR-21 (12), which were identified to be differentially expressed in ALD patients by microarray, were also identified as differentially expressed in ALD in this animal model study. However, further verification in an independent human setting and on a large scale is required. The present study does not preclude the possibility that those miRNAs with significantly modified expression levels appeared in liver tissues prior to hepatic lipid accumulation. Therefore, conducting a rat model analysis over 6 or ~10 weeks requires to be considered in subsequent studies. The difficulties in performing liver biopsies in ALD patients may reduce the application of miRNAs in ALD. However, the emergence of serum miRNA may solve this problem and function as a non-invasive diagnostic tool in ALD.

In conclusion, to the best of our knowledge, the present study provided miRNA profiles at different stages of ALD for the first time in a rat model, with satisfactory diagnostic values. In addition, the miRNA-pair reservoir obtained may broaden the understanding of ALD and become a potential basis for diagnosis and therapeutic interventions.

## Figures and Tables

**Table I:** Change of hepatic and serologic markers in different stages of alcoholic liver disease.

	Group			
	
	ASH	C16	AFL	C12
Hepatic Index (%)	4.33±0.21^a^	3.02±0.17	3.99±0.17^c^	2.89±0.12
ALT (IU/l)	153.69±19.34^b^	57.46±11.23	62.33±10.05	58.59±12.07
AST (IU/l)	271.78±39.16^a^	156.34±12.25	143.69±23.15	151.27±17.39
TG (mmol/l)	0.91±0.07^a^	0.63±0.11	1.58±0.08^c^	0.59±0.09
TCh (mmol/l)	3.39±0.18^a^	1.46±0.09	4.57±0.22^c^	1.38±0.15
GGT (IU/l)	143.12±15.69^b^	35.78±8.27	89.54±11.47^c^	31.87±9.56
Hepatic TG (mmol/l)	5.37±0.69^a^	1.56±0.16	5.11±0.75^c^	1.43±0.13
HAI score	3.57±0.44^a^	1.21±0.17	1.54±0.39	1.33±0.18

Values are expressed as mean ± standard error of the mean.

a P<0.05,

b P<0.01, compared with the C16 group;

c P<0.05, compared with the C12 group.

ASH, alcoholic steatohepatitis group; C16, normal 16-week liver group; AFL, simple steatosis group; C12, normal 12-week liver group; ALT, alanine aminotransferase; AST, aspartate aminotransferase; TG, triglyceride; TCh, total cholesterol; GGT, gamma-glutamic transpeptidase; HAI, histological activation index.

**Table II:** Significantly dysregulated miRNAs in liver tissue of a rat model of alcoholic liver disease confirmed by quantitative polymerase chain reaction.

ASH/C16		AFL/C12	
	
miRNA	Fold change	miRNA	Fold change
rno-miR-129	12.80	rno-miR-200c^*^	3.26
rno-miR-3586-5p	10.10	rno-miR-410^*^	3.03
rno-miR-487b^*^	6.66	rno-miR-490	2.99
rno-miR-2985	4.86	rno-miR-195^*^	2.23
rno-miR-344a-3p	4.05	rno-miR-466d	1.67
rno-miR-299	3.53	rno-miR-93	**0.18**
rno-miR-490	3.11	rno-miR-221^*^	**0.22**
rno-miR-667	2.98	rno-miR-451	**0.32**
rno-miR-21^*^	2.74	rno-miR-146a	**0.33**
rno-miR-30b-3p	2.66	rno-miR-191	**0.38**
rno-miR-503	2.53	rno-miR-17-5p	**0.42**
rno-miR-183	2.50	rno-miR-140^*^	**0.46**
rno-miR-185^*^	2.36	rno-miR-7a	**0.55**
rno-miR-329^*^	2.01		
rno-miR-291a-5p	1.71		
rno-miR-34b^*^	1.50		
rno-miR-199a-3p	**0.12**		
rno-miR-214	**0.17**		
rno-miR-181a	**0.18**		
rno-miR-93	**0.28**		
rno-miR-146a	**0.34**		
rno-miR-151	**0.35**		
rno-let-7b	**0.39**		
rno-miR-140^*^	**0.39**		
rno-miR-148b-3p	**0.45**		
rno-miR-451	**0.49**		
rno-miR-191	**0.52**		
rno-miR-31	**0.55**		
rno-miR-7a	**0.55**		

Downregulation is shown in bold. miRNA, microRNA; ASH, alcoholic steatohepatitis group; C16, normal 16-week liver group; AFL, simple steatosis group; C12, normal 12-week liver group.

**Table III:** Significance analysis of microarray results of significantly dysregulated miRNAs in alcoholic liver disease.

ASH/C16		AFL/C12	
	
miRNA	Fold Change	miRNA	Fold Change
rno-miR-129	12.81	rno-miR-200c^*^	3.26
rno-miR-344a-3p	4.05	rno-miR-410^*^	3.03
rno-miR-299	3.53	rno-miR-490	2.99
rno-miR-490	3.11	rno-miR-195^*^	2.23
rno-miR-21^*^	2.74	rno-miR-93	**0.18**
rno-miR-30b-3p	2.66	rno-miR-221^*^	**0.22**
rno-miR-503	2.53	rno-miR-451	**0.32**
rno-miR-183	2.50	rno-miR-146a	**0.33**
rno-miR-185^*^	2.36	rno-miR-191	**0.38**
rno-miR-329^*^	2.01	rno-miR-17-5p	**0.42**
rno-miR-291a-5p	1.71	rno-miR-140^*^	**0.46**
rno-miR-199a-3p	**0.12**		
rno-miR-214	**0.17**		
rno-miR-181a	**0.18**		
Rno-miR-93	**0.28**		
rno-miR-146a	**0.34**		
rno-miR-151	**0.35**		
rno-let-7b	**0.39**		
rno-miR-140^*^	**0.39**		
rno-miR-451	**0.49**		
rno-miR-191	**0.52**		

Downregulation is shown in bold. miRNA, microRNA; ASH, alcoholic steatohepatitis group; C16, normal 16-week liver group; AFL, simple steatosis group; C12, normal 12-week liver group.

**Table IV:** Top five genes targeted by all dysregulated micro RNAs.

Group	Regulation	Gene ID	Gene description
ASH	Down	Eltd1	EGF, latrophilin and seven transmembrane domain containing 1
		Prkx	Protein kinase, X-linked
		Bdnf	Brain-derived neurotrophic factor
		Snap29	Synaptosomal-associated protein 29
		Padi2	Peptidyl arginine deiminase
	Up	Aqp1	Aquaporin 1
		Nr3c1	Nuclear receptor subfamily 3, group C, member 1
		Slc25a27	Solute carrier family 25, member 27
		Tnfrsf8	Tumor necrosis factor receptor superfamily, member 8
		Ppt2	Palmitoyl-protein thioesterase 2
AFL	Down	Wasf1	WAS protein family, member 1
		Eif4a2	Eukaryotic translation initiation factor 4A2
		Nr4a3	Nuclear receptor subfamily 4, group A, member 3
		Prps2	Phosphoribosyl pyrophosphate synthetase 2
		RGD1307799	Increased sodium tolerance 1 homolog
	Up	Wif1	Wnt inhibitory factor 1
		Dync1li2	Dynein, cytoplasmic 1 light intermediate chain 2
		Slc16a7	Monocarboxylic acid transporter 2
		Trhr	Thyrotropin releasing hormone receptor
		Chrdl1	Chordin-like 1

The top five target genes were screened according to the miRNA/target gene pair-specific context score. ASH, alcoholic steatohepatitis group; C16, normal 16-week liver group; AFL, simple steatosis group; C12, normal 12-week liver group.

**Table V:** Top five Gene Ontology term enrichment analysis of the target genes of downregulated or upregulated microRNAs.

Group	Regulation	Term	Function	P-value	Genes
ASH	Down	GO:0022900	Electron transport chain	0.035	CYBASC3, BDNF
		GO:0016310	Phosphorylation	0.045	BDNF, SYK, PRKX
		GO:0003001	Generation of a signal involved in cell-cell signaling	0.052	BDNF, SYK
		GO:0006796	Phosphate metabolic process	0.061	BDNF, SYK, PRKX
		GO:0006793	Phosphorus metabolic process	0.061	BDNF, SYK, PRKX
		GO:0032940	Secretion by cell	0.098	BDNF, SYK
ASH	Up	GO:0007242	Intracellular signaling cascade	0.002	GDI1, DAB2IP, RASSF5, MAP2K1, MRAS, ARF3, RAB15, TGFA, RHOQ, NR3C1, TSHR
		GO:0010033	Response to organic substance	0.002	STAR, MAP2K1, P2RX3, RHOQ, NR3C1, AKAP1, AQP1, AK3L1, KCNJ11, CPT1A, IL6RA
		GO:0009719	Response to endogenous stimulus	0.006	STAR, MAP2K1, RHOQ, AKAP1, AQP1, AK3L1, KCNJ11, IL6RA
		GO:0006812	Cation transport	0.010	SLC4A10, SLC12A2, SLC24A2, P2RX3, KCNIP2, AQP1, KCNJ11, KCHIP2
AFL	Up	GO:0016337	Cell-cell adhesion	7.98×10^−8^	PCDHA6, PCDHA7, PCDHA8, PCDHA9, OLR1, PCDHA2, PCDHA3, PCDHA4, PCDHA5, PCDHA1, CD164, PCDHAC2, PCDHAC1, NRCAM, PCDHA10, PCDHA11, LOC317356, PCDHA12, PCDHA13, SELE
		GO:0007156	Homophilic cell adhesion	5.24×10^−7^	PCDHA6, PCDHA7, PCDHA8, PCDHA9, PCDHA2, PCDHA3, PCDHA4, PCDHA5, PCDHA1, PCDHAC2, PCDHAC1, PCDHA10, LOC317356, PCDHA11, PCDHA12, PCDHA13
		GO:0022610	Biological adhesion	6.85×10^−5^	PCDHA6, PCDHA7, PCDHA8, PCDHA9, OLR1, PCDHA2, PCDHA3, PCDHA4, PCDHA5, KITLG, PCDHA1, CD164, PCDHAC2, PCDHAC1, NRCAM, PCDHA10, PCDHA11, LOC317356, PCDHA12, PCDHA13, SELE
		GO:0007155	Cell adhesion	6.85×10^−5^	PCDHA6, PCDHA7, PCDHA8, PCDHA9, OLR1, PCDHA2, PCDHA3, PCDHA4, PCDHA5, KITLG, PCDHA1, CD164, PCDHAC2, PCDHAC1, NRCAM, PCDHA10, PCDHA11, LOC317356, PCDHA12, PCDHA13, SELE
		GO:0007264	Small GTPase-mediated signal transduction	0.001	ARL5A, RGD1566257, RABIF, RGD1563962, RRAS2, MRAS, RAB35, RAF1, RHOQ, ARL8B, ARHGDIA

The top five gene ontology terms were screened according to the P-value. ASH, alcoholic steatohepatitis group; C24, normal 24-week liver group; AFL, simple steatosis group; C12, normal 12-week liver group; GTPase, guanosine triphosphatase.

**Table VI:** Kyoto Encyclopedia of Genes and Genomes pathway enrichment analysis for the target genes of downregulated or upregulated microRNAs.

Group	Regulation	Term	Function	P-value	Genes
ASH	Up	rno05200	Signaling pathways in cancer	0.013948	RASSF5, MAP2K1, FZD1, SMAD3, TGFA, RBX1
		rno05223	Non-small cell lung	0.023922	RASSF5, MAP2K1, TGFA cancer
		rno05212	Pancreatic cancer	0.040337	MAP2K1, SMAD3, TGFA
		rno05211	Renal cell carcinoma	0.040337	MAP2K1, TGFA, RBX1
		rno05210	Colorectal cancer	0.053856	MAP2K1, FZD1, SMAD3
AFL	Up	rno05210	Colorectal cancer	0.006536	TGFBR1, FZD1, RAF1, SMAD3, MAPK9
		rno04010	MAPK signaling pathway	0.010884	RGD1566257, RRAS2, MRAS, TGFBR1, NTRK2, RAF1, MAPK9, PPM1B, CACNA1C
		rno05212	Pancreatic cancer	0.025860	TGFBR1, RAF1, SMAD3, MAPK9
		rno05200	Signaling pathways in cancer	0.026197	RASSF5, VHL, TGFBR1, FZD1, RAF1, SMAD3, KITLG, MAPK9
		rno04722	Neurotrophin signaling pathway	0.028982	NTRK2, RAF1, MAPK9, YWHAE, ARHGDIA
		rno04310	Wnt signaling pathway	0.045010	SIAH1A, FZD1, SMAD3, MAPK9, WIF1
		rno04142	Lysosome	0.094444	SLC11A2, PLA2G15, IGF2R, CD164
		rno04080	Neuroactive ligand-receptor interaction	0.094769	P2RX3, GLRA2, TRHR, P2RX2, NR3C1, TSHR

ASH, alcoholic steatohepatitis group; C16, normal 16-week liver group; AFL, simple steatosis group; C12, normal 12-week liver group; MAPK, mitogen-activated protein kinase.
